# Favorable long-term clinical and radiologic outcomes with high survivorship after autologous osteochondral transplantation of the talus

**DOI:** 10.1007/s00167-022-07237-3

**Published:** 2022-11-17

**Authors:** Philipp W. Winkler, Stephanie Geyer, Daniela Walzl, Klaus Woertler, Jochen Paul, Sebastian Siebenlist, Andreas B. Imhoff, Andrea Achtnich

**Affiliations:** 1 Department of Sports Orthopaedics, Klinikum Rechts Der Isar, Technical University of Munich, Ismaninger Str. 22, 81675 Munich, Germany; 2 Musculoskeletal Radiology Section, Klinikum Rechts Der Isar, Technical University of Munich, Munich, Germany; 3 Rennbahnklinik, Muttenz, Switzerland

**Keywords:** Osteochondral lesion, OATS, Cartilage, Autograft, Survival, MOCART

## Abstract

**Purpose:**

To evaluate long-term clinical and radiologic outcomes of patients undergoing autologous osteochondral transplantation (AOT) for osteochondral lesions of the talus (OLT) and to perform a correlation analysis between clinical and radiologic outcomes.

**Methods:**

Thirty-five patients with a mean age of 32.2 ± 8.9 years undergoing AOT for OLT between 1997 and 2003 were available for follow-up after an average of 19.1 ± 1.4 years. Demographic, surgical, and injury-related data were collected. After a minimum 18-year follow-up, patient-reported outcome scores (PROs) were collected, including the American Orthopaedic Foot & Ankle Society (AOFAS) score, the Foot and Ankle Outcome Score (FAOS), Tegner Activity Scale, and Visual Analogue Scale (VAS) for pain of the ankle. The Lysholm Score and VAS for pain of the knee were collected to assess donor-site morbidity. Magnetic resonance imaging scans were obtained to conduct an assessment of the replaced cartilage using the Magnetic Resonance Observation of Cartilage Repair Tissue (MOCART) 2.0 scoring system. Any revision surgery (except symptomatic hardware removal and arthroscopic debridement) was defined as clinical failure.

**Results:**

Favorable clinical and radiologic (MOCART score, 73.7 ± 16.7 points) outcomes without any donor-site morbidities were observed. Twenty-three (65.7%) patients were satisfied or very satisfied with the surgical treatment. Fourteen (40.0%) and 25 (71.4%) patients had no or minor limitations in their athletic and working performance, respectively. A significant correlation between the MOCART and the FAOS Sport and Recreational activities subscale was found (*r*_*s*_, 0.491; *p* = 0.033). Six (17.1%) patients met the criteria for clinical failure an average of 12.2 ± 6.6 years after AOT. Survival analysis demonstrated a mean estimated time of survival of 21.3 years (95% CI [19.55, 22.96]) and a 20-year survival rate of 77.9%.

**Conclusion:**

Autologous osteochondral transplantation to treat OLT achieves high patient satisfaction and favorable PROs with a 20-year survival rate of almost 80%. Given the high clinical efficacy of AOT, this procedure can be recommended as a safe and promising technique for the long-term therapy of OLT.

**Level of evidence:**

Level IV.

## Introduction

Non-operative treatment of osteochondral lesions of the talus (OLT) is often considered as first-line therapy [28]. However, persistent pain, locking symptoms, and progression of ankle osteoarthritis (OA) affect the quality of life and, in particular, physical and athletic performance. In one study, 58%, 23%, and 10% of patients with OLT undergoing non-operative treatment reported limitations in sports, activities of daily living, and work, respectively [11]. Consequently, in patients with OLT and failed non-operative treatment, surgical treatment should be considered to prevent early ankle deterioration and achieve satisfactory long-term outcomes [3].

There is controversy regarding the ideal technique for cartilage repair/replacement in focal OLT. Bone marrow stimulating (BMS) techniques are simple, cost-effective, and minimally invasive single-stage procedures to treat OLT [6, 15, 17]. However, BMS techniques are not recommended in cases of extensive subchondral bone damage or in OLT greater than 107 mm^2^ in area and/or 10 mm in diameter [19]. There is compelling evidence for autologous chondrocyte implantation (ACI) in the treatment of OLT, with recent systematic reviews reporting clinical success rates of almost 90% [9, 16]. However, ACI is a two-stage procedure associated with higher costs and increased discomfort for the patient [15]. Autologous osteochondral transplantation (AOT) represents a single-stage procedure to treat OLT. Transplantation of autologous osteochondral dowels including hyaline cartilage, subchondral bone plate, and cancellous bone, restores a biomechanically stable and native articular environment [15]. Good to excellent short- and mid-term clinical outcomes after AOT for OLT have been reported in up to 87% of patients with reoperation and failure rates as low as 6% and 1%, respectively [7, 24]. However, long-term results are of high clinical relevance to assess the longevity of this procedure and are still pending [14].

The objective of this study was to evaluate long-term clinical, functional, and radiologic outcomes of patients undergoing AOT for OLT and to perform a correlation analysis between clinical and radiologic outcomes. It was hypothesized that patients undergoing AOT would achieve satisfactory clinical and radiologic long-term outcomes based on patient-reported outcomes (PRO) and the Magnetic Resonance Observation of Cartilage Repair Tissue (MOCART) 2.0 scoring system, respectively.

### Material and methods

This study was approved by the Institutional Review Board of the Technical University of Munich (No.: 53/20 S-KH). All included patients signed a written informed consent form to participate in the study.

Patients undergoing AOT for OLT between 1997 and 2003 at the authors’ institution were screened for eligibility for this retrospective case series. Inclusion criteria included: 16–60 years of age at the time of index procedure, minimum follow-up of 18 years, single-stage AOT for focal OLT, and ipsilateral knee as the graft harvest site. Patients with a history of previous ankle surgery were also included. Patients presenting with end-stage OA of the ankle, multiple OLT, bipolar lesions, bony ankle deformity, chronic instability, and a history of previous distal tibia, fibular, or talar fracture were excluded from the study.

#### Indications and surgical technique

AOT was performed in patients with chronic ankle pain due to OLT (> 10 mm in diameter, < 200 mm^2^) who had failed non-operative treatment for at least 12 weeks. For centrally and posteriorly located OLT, an osteotomy of the medial malleolus or the anterolateral tibia (i.e., Tillaux-Chaput Tubercle) was performed. All procedures were performed using the Osteochondral Autograft Transfer System (OATS®, Arthrex Inc., Naples, FL, USA). A lateral mini-arthrotomy was performed on the ipsilateral knee to harvest a size-matched osteochondral dowel from the proximal-lateral trochlea using the donor harvester. Finally, the graft was fixed in the OLT using a press-fit technique. Osteotomies were fixed using lag screws. Non-weight bearing, continuous passive motion exercises, and a protective ankle brace were recommended for 6 weeks postoperatively. Afterwards, weight bearing as tolerated was permitted [21]. Return to low- and high-impact sports was permitted three and six months after AOT, respectively.

#### Demographic, surgical, and injury-related data

A retrospective chart review was performed to collect demographic and surgical data of eligible patients. Demographic data included: age and body mass index (BMI) at the time of index procedure, sex, laterality, smoking status (yes, no), trauma history of the index ankle (yes, no), and surgical history. Surgical data included: location of the OLT, size of OLT, number of osteochondral dowels transplanted, need for osteotomy during the surgical approach, and subsequent surgical procedures.

According to previous research, the location of OLT was classified using a 9-zone grid [4, 26]. Based on the diameter of the osteochondral dowels transplanted, the size of the OLT was calculated like the area of a circle. In cases where more than one dowel was transplanted, the areas of the corresponding circles were added to determine the size of the OLT.

#### Clinical outcomes

At final follow-up, a standardized clinical examination of the lower extremities, including the knee and ankle joints, was performed. Knee and hindfoot alignment were assessed clinically. Ankle range of motion (ROM) was defined as the maximum passive arc of motion from dorsiflexion to plantarflexion. Standardized and validated PROs were collected to assess functional outcomes of the ankle, including the American Orthopaedic Foot & Ankle Society (AOFAS) score, the Foot and Ankle Outcome Score (FAOS), Tegner Activity Scale, and Visual Analogue Scale (VAS) for pain of the ankle. The Lysholm Score and VAS for pain of the knee were collected to assess donor-site morbidity.

The type, frequency (hours per week), and limitations (no limitation, minor limitation, major limitation) in athletic performance were recorded. In addition, the type of employment (full-time, part-time, housekeeping, retired, disabled), limitations in working performance (no limitation, minor limitation, major limitation, disabled), and the level of satisfaction (very satisfied, satisfied, unsatisfied, very unsatisfied) with the surgical treatment were surveyed.

#### Magnetic resonance (MR) imaging

Magnetic resonance (MR) imaging was performed on a 3 Tesla whole-body scanner (Verio, Siemens, Erlangen, Germany) with use of an 8-channel head coil. The following pulse sequences were acquired: sagittal and coronal intermediate-weighted turbo spin echo (TSE) sequences (BLADE) with spectral fat saturation (echo train length [ETL], 9; repetition time [TR], 4500 ms; echo time [TE], 46 ms; field-of-view [FOV], 140 mm; in-plane resolution, 0.4 × 0.4 mm; slice thickness [ST], 3 mm) and sagittal and coronal T1-weighted TSE sequence with a driven equilibrium (DRIVE) pulse for native arthrographic contrast (ETL, 3; TR, 1000 ms; TE, 13 ms; FOV, 140 mm; in-plane resolution, 0.4 × 0.4 mm; ST, 3 mm). Magnetic resonance images of the affected ankles were acquired with the patient in supine position and neutral ankle flexion and neutral tibial rotation.

**Fig. 1:**
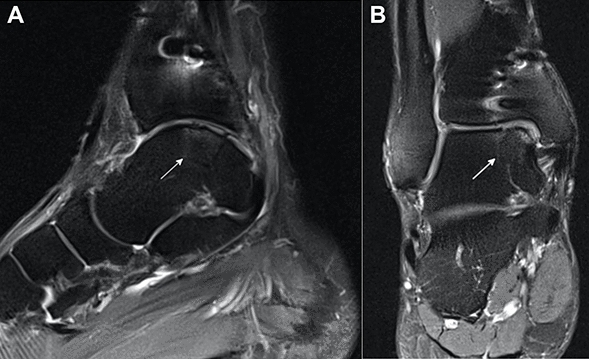
MRI assessment of the replaced cartilage. Sagittal (**A**) and coronal (**B**) intermediate-weighted turbo spin echo images of the right ankle of a 26-year-old male patient (at the time of AOT) with a MOCART 2.0 score of 85 points at 19.3-years follow-up. The patient was treated with AOT (1 osteochondral dowel, white arrow) combined with an osteotomy of the medial malleolus. *AOT* Autologous osteochondral transplantation; *MOCART* Magnetic Resonance Observation of Cartilage Repair Tissue

At final follow-up, MR scans were obtained to perform a standardized and validated assessment of the replaced cartilage tissue using the MOCART 2.0 scoring system (Fig. 1) [1, 2, 22]. The MOCART 2.0 score was independently obtained by one musculoskeletal radiologist with more than 20 years of experience (KW) and two orthopedic surgeons (PWW, SG) using a Picture Archiving and Communication System (PACS). All three observers were blinded to each other’s results. One observer (PWW) obtained the MOCART 2.0 score for all patients three times at 2-week intervals. Intraclass correlation coefficients (ICC) were calculated to assess intra- and interrater reliability. There was excellent intra- (ICC, 0.988 95% CI [0.974, 0.995]) and interrater (ICC, 0.965 95% CI [0.925, 0.985]) reliability in the assessment of the replaced cartilage tissue based on the MOCART 2.0 scoring system.

#### Clinical failure

Any revision surgery (except symptomatic hardware removal and arthroscopic debridement) due to persistent/recurrent pain, discomfort, or dysfunction of the index ankle was defined as clinical failure of AOT [18, 23]. The time between AOT and clinical failure or revision surgery and the type of revision surgery were recorded.

#### Statistical analysis

Categorical variables were reported as number of patients and corresponding percentage. The Shapiro–Wilk test was used to assess the distribution of continuous variables. Normally distributed variables were reported as mean, standard deviation, and range. Non-normally distributed variables were reported as median and inter-quartile range (IQR). A Spearman’s rank-order correlation analysis was conducted to assess the relationship between radiologic (MOCART 2.0 scoring system) and clinical outcomes (FAOS subscales, AOFAS score, VAS for pain, Tegner Activity Scale, Lysholm Score, sports frequency, ankle ROM) in patients undergoing AOT for OLT. The Kaplan–Meier method was applied to evaluate the mean estimated time of survival. Clinical failure was defined as the endpoint for the survival analysis. SPSS software version 26.0 (IBM-SPSS, New York, USA) was used for statistical analysis and the level of significance was set at *p* < 0.05.

### Results

**Fig. 2:**
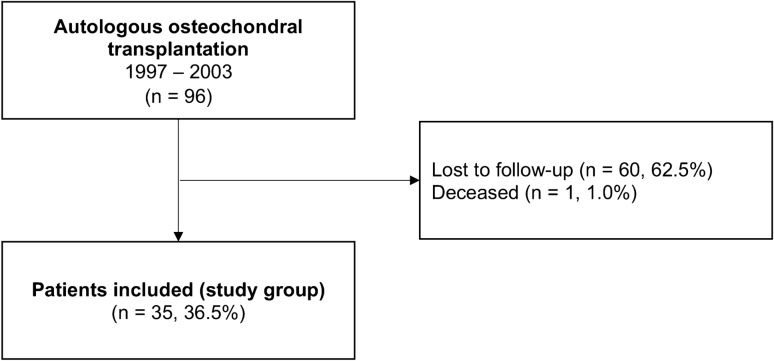
Flowchart of patient recruitment. *n*, number of patients and corresponding percentage

During the 6-year period screened for this study, 96 patients underwent AOT for the treatment of OLT. Sixty patients (62.5%) were lost to follow-up (change of residence or contact details) and one patient deceased (1.0%) during the follow-up period. Consequently, 35 (36.5%) patients with a mean age of 32.2 ± 8.9 years at the time of the index procedure were available for final follow-up after an average of 19.1 ± 1.4 years (Fig. 2). In 30 (85.7%) patients, AOT was performed in conjunction with an osteotomy of the medial malleolus or the anterolateral tibia. The mean lesion size was 124.0 ± 44.1 mm^2^, which was treated with one and two osteochondral dowels in 14 (40.0%) and 21 (60.0%) patients, respectively. Demographic, surgical, and injury-related data are shown in detail in Table 1.

**Table 1:** Baseline characteristics

Variable	Total study group
Number of included patients, *n*	35
Age [years]	32.2 ± 8.9 (17–50)
BMI [kg/m^2^]^a^	26.4 (7.6)
Follow-up [years]	19.1 ± 1.4 (18.0–23.4)
Lesion size [mm^2^]	124.0 ± 44.1 (50.3–190.1)
Male, *n* (%)	19 (54.3)
Right ankle, *n* (%)	17 (48.6)
Smoker, *n* (%)	8 (22.9)
Previous surgery, *n* (%)	11 (31.4)
Type previous surgery	
Debridement, *n* (%)	5 (45.5)
Microdrilling, *n* (%)	5 (45.5)
Stabilization, *n* (%)	1 (9.1)
Affected talar zone^b^	
Zone 1, *n* (%)	2 (5.7)
Zone 2, *n* (%)	0
Zone 3, *n* (%)	0
Zone 4, *n* (%)	21 (60.0)
Zone 5, *n* (%)	1 (2.9)
Zone 6, *n* (%)	5 (14.3)
Zone 7, *n* (%)	10 (28.6)
Zone 8, *n* (%)	0
Zone 9, *n* (%)	1 (2.9)

Continuous variables are presented as mean ± standard deviation (range), unless otherwise noted. Categorical variables are presented as number (percentage of total study group)

*BMI* body mass index

^a^Median (inter-quartile range)

^b^The cumulative percentage exceeds 100% as some osteochondral lesions affected multiple zones

Given the high drop-out rate (63.5%) during the minimum 18-year follow-up period, a drop-out analysis was conducted using the available demographic data. No differences in age (32.2 ± 8.9 years vs. 30.7 ± 7.4 years, *p* > 0.05), sex (54% male vs. 66% male, *p* > 0.05), and laterality (51% left vs. 51% left, *p* > 0.05) were observed between included patients and patients lost to follow-up.

#### Clinical and radiologic outcomes

**Fig. 3:**
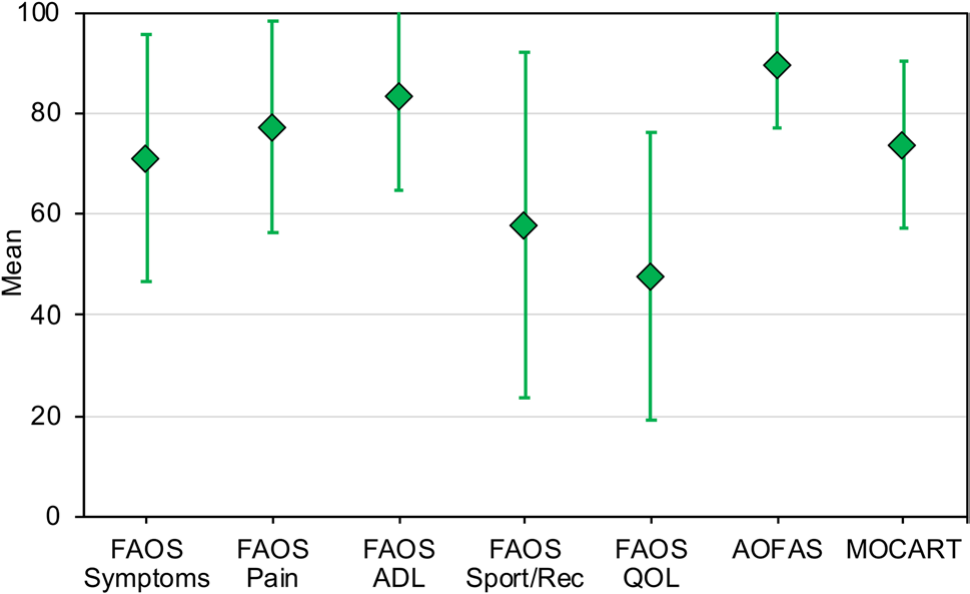
Ankle-related scores. Means and standard deviations are represented by the rhombuses and error bars, respectively. *ADL* Activities of Daily Living; *AOFAS* American Orthopaedic Foot & Ankle Society score; *FAOS* Foot and Ankle Outcome Score; *MOCART* Magnetic Resonance Observation of Cartilage Repair Tissue 2.0 scoring system; *QOL* Foot and Ankle-Related Quality of Life; *Sport/Rec* Sport and Recreational activities

Patient-reported outcomes were collected in all patients of the study group (100%), while only twenty patients (57.1%) agreed to undergo MRI examination. Patient-reported outcomes evaluating ankle function and donor-site morbidity are displayed in Table 2. Analysis of the MR scans revealed a mean MOCART 2.0 score of 73.7 ± 16.7 points (range, 40–100 points). Ankle-related scores are displayed graphically in Fig. 3. No malalignment could be detected during clinical examination. The mean ankle ROM was 54.9 ± 13.9 degrees (range, 30.0–80.0 degrees). At the final follow-up, 23 (65.7%) patients were satisfied or very satisfied with the surgical treatment. Moreover, 14 (40.0%) and 25 (71.4%) patients had no or minor limitations in their athletic and working performance, respectively. More details regarding sports and work are outlined in Table 3.

**Table 2:** Ankle and donor-site related patient-reported outcomes

Variable	Total study group
FAOS	
Symptoms [points]	71.0 ± 24.5 (2–100)
Pain [points]	77.3 ± 21.0 (39–100)
ADL [points]	83.3 ± 18.5 (46–100)
Sport/Rec [points]	57.7 ± 34.3 (0–100)
QOL [points]	47.8 ± 28.6 (6–100)
AOFAS [points]	89.6 ± 12.5 (55–100)
VAS for pain ankle [points]	3.1 ± 2.9 (0–8.1)
Tegner Activity Scale [points]^a^	3 (2)
Lysholm Score [points]	82.2 ± 21.6 (0–100)
VAS for pain Knee [points]	0.9 ± 1.1 (0–3.3)

Continuous variables are presented as mean ± standard deviation (range), unless otherwise indicated

*ADL* Activities of Daily Living; *AOFAS* American Orthopaedic Foot & Ankle Society score; *FAOS* Foot and Ankle Outcome Score; *QOL* Foot and Ankle-Related Quality of Life; *Sport/Rec* Sport and Recreational activities; *VAS* Visual Analogue Scale

^a^Median (inter-quartile range)

**Table 3:** Athletic and working performance

Variable	Total study group
Primary sport	
None, *n* (%)	5 (14.3)
Swim, *n* (%)	3 (8.6)
Hiking, *n* (%)	1 (2.9)
Bike, *n* (%)	9 (25.7)
Ski, *n* (%)	2 (5.7)
Fitness/gym, *n* (%)	9 (25.7)
Climb, *n* (%)	1 (2.9)
Run, *n* (%)	2 (5.7)
Soccer, *n* (%)	3 (8.6)
Sports frequency [h/week]	3.6 ± 4.6 (0–21.0)
Limitations in athletic performance	
No limitation, *n* (%)	6 (17.0)
Minor limitation, *n* (%)	8 (22.9)
Major limitation, *n* (%)	21 (60.0)
Employment type	
Full-time, *n* (%)	23 (65.7)
Part-time, *n* (%)	6 (17.1)
Housekeeping, *n* (%)	3 (8.6)
Retired, *n* (%)	1 (2.9)
Disabled, *n* (%)	2 (5.7)
Limitations in working performance	
No limitation, *n* (%)	22 (62.9)
Minor limitation, *n* (%)	3 (8.6)
Major limitation, *n* (%)	6 (17.1)
Disabled, *n* (%)	4 (11.4)
Satisfaction	
Very satisfied, *n* (%)	10 (28.6)
Satisfied, *n* (%)	13 (37.1)
Unsatisfied, *n* (%)	4 (11.4)
Very unsatisfied, *n* (%)	8 (22.9)

Continuous variables are presented as mean ± standard deviation (range). Categorical variables are presented as number (percentage of total study group)

A statistically significant and positive correlation between the MOCART 2.0 score and the FAOS Sport and Recreational activities (Sport/Rec) subscale could be detected (*r*_*s*_, 0.491; *p* = 0.033). More details of the correlation analysis are shown in Table 4.

**Table 4:** Correlation analysis between clinical and radiologic outcomes

	MOCART score
FAOS Symptoms	*r*_*s*_ = 0.055 *n.s*
FAOS Pain	*r*_*s*_ = 0.274 *n.s*
FAOS Sport/Rec^a^	*r*_*s*_ = 0.491 *p* = 0.033
FAOS ADL	*r*_*s*_ = 0.209 *n.s*
FAOS QOL	*r*_*s*_ = 0.173 *n.s*
AOFAS	*r*_*s*_ = 0.029 *n.s*
VAS for pain ankle	*r*_*s*_ = – 0.152 *n.s*
VAS for pain knee	*r*_*s*_ = – 0.076 *n.s*
Lysholm Score	*r*_*s*_ = 0.276 *n.s*
Tegner Activity Scale	*r*_*s*_ = 0.237 *n.s*
Ankle range of motion	*r*_*s*_ = 0.440 *n.s*
Sports frequency	*r*_*s*_ = – 0.007 *n.s*

*ADL* Activities of Daily Living; *AOFAS* American Orthopaedic Foot & Ankle Society ankle-hindfoot score; *FAOS* Foot and Ankle Outcome Score; *MOCART* Magnetic Resonance Observation of Cartilage Repair Tissue 2.0 scoring system; *n.s.* non-significant; *p* p-value; *QOL* Foot and Ankle-Related Quality of Life; *r*_*s*_ Spearman’s correlation; *Sport/Rec* Sport and Recreational activities; *VAS* Visual Analogue Scale

^a^Statistically significant positive correlation

#### Revision surgery, clinical failure, and survivorship

An average time of 2.6 ± 5.2 years (range, 0.2–18.0 years) after AOT, 25 (71.4%) patients underwent revision surgery, including symptomatic hardware removal (*n* = 24), arthroscopic debridement (*n* = 3), revision AOT (*n* = 2), ankle arthrodesis (*n* = 2), bone grafting (*n* = 1), and Autologous Matrix-Induced Chondrogenesis (AMIC; *n* = 1). In total, 6 (17.1%) patients met the criteria for clinical failure an average of 12.2 ± 6.6 years (range, 4.0–19.0 years) after AOT. No significant differences could be observed between patients with vs. without clinical failure regarding baseline characteristics, clinical and radiologic outcomes (all, *p* > 0.05). Moreover, no donor-site morbidities were reported.

**Fig. 4:**
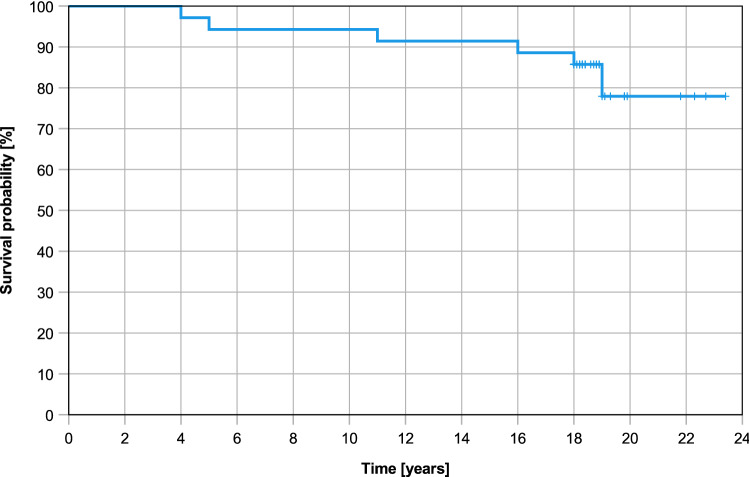
Kaplan–Meier survival analysis. Clinical failure (any revision surgery, except symptomatic hardware removal and arthroscopic debridement) was defined as the endpoint

The survival analysis demonstrated a mean estimated time of survival of 21.3 years (95% CI [19.55, 22.96]) for AOT in the treatment of OLT. Additionally, AOT survival was found to be 94.3%, 94.3%, 91.4%, and 77.9% at 5, 10, 15, and 20 years, respectively (Fig. 4).

## Discussion

The most important finding of this study was that AOT is a safe and viable technique to treat OLT, achieving favorable long-term clinical outcomes with an acceptable rate of limitations in activities of daily living and athletic and working performance. A 20-year survival rate of almost 80% underscores the high clinical efficacy of AOT. Another pivotal finding was a significant and positive correlation between the MR imaging appearance of the replaced cartilage and the FAOS Sport/Rec subscale, supporting surgeons in counseling patients regarding sports activities after AOT.

In a study investigating 189 patients undergoing primary arthroscopic BMS for OLT, significant improvements in VAS for pain and AOFAS score were found after an average of 13.9 years. Despite satisfactory functional outcomes, the authors noted a significant deterioration in both scores from short- and mid-term to long-term follow-ups [17]. Satisfactory long-term clinical and functional outcomes after arthroscopic BMS were confirmed in a recent systematic review [20]. However, survival analysis showed that patients undergoing arthroscopic BMS in OLT of ≥ 150 mm^2^ in size had a significantly lower survival rate than patients with smaller OLT at mid- and long-term follow-up [17, 23]. Therefore, other surgical procedures such as AOT are recommended in the treatment of larger OLT. In this study, a mean AOFAS score of 89.6 ± 12.5 points after an average of 19.1 years after AOT for the treatment of OLT is comparable to the reported studies. However, patients undergoing AOT in this study had a mean lesion size of 124 mm^2^, while studies evaluating BMS and ACI for the treatment of OLT reported a mean lesion size of 100–105 mm^2^ and 198–204 mm^2^, respectively [12, 13, 17, 20]. Consequently, the available data indicate that BMS, ACI, and AOT may result in satisfactory long-term clinical and functional outcomes if properly indicated depending on the size of the OLT.

Autologous osteochondral transplantation in the treatment of OLT has been shown to result in high patient satisfaction at mid-term follow-up. An average of 7 years after AOT, 25 patients reported favorable PROs, resulting in 88% of patients being satisfied or very satisfied [10]. Good mid-term results were confirmed by another study after 18 primary AOT of the talus and a mean follow-up of 7.6 years [23]. These promising results are in line with a systematic review including 500 patients with a mean weighted follow-up of 5.2 years [24]. However, long-term follow-up studies after AOT for the treatment of OLT are lacking. There are hardly any studies reporting a minimum or even mean follow-up period of more than 10 years. In one of the longest reported follow-ups after AOT for OLT, 20 patients showed good results regarding the AOFAS score, VAS for pain, and Lysholm Score after a mean follow-up of 12 years (minimum follow-up, 10 years) [25]. In this study, the minimum follow-up was 18 years, and the PROs are comparable to the mid-term results of previous studies. Consequently, restoration of the biomechanical properties of the native cartilage-bone unit by AOT appears to be a viable treatment modality for the management of OLT that maintains favorable outcomes for up to 20 years.

The clinical failure rate after AOT ranges from 1 to 24% after 5–8 years of follow-up [18, 23, 24]. Although some studies have reported risk factors for clinical failure, such as larger lesion size or secondary AOT compared with primary AOT, no consistent factors have yet been identified [10, 18]. In this study, no demographic, surgical, or injury-related characteristics were associated with clinical failure resulting in a survival rate of 77.9% at 20 years and a mean estimated time of survival of 21.3 years.

The correlation between clinical and radiologic outcomes after cartilage restoration procedures of the talus remains controversial. No correlation between the MOCART score and clinical outcomes could be observed after matrix-induced ACI and AMIC for the treatment of OLT after a mean follow-up of 12 years and 4.7 years, respectively [13, 27]. Similarly, one study showed no correlation between MR imaging findings and clinical outcomes after AOT for OLT [10]. In this study, a significant and positive correlation between the MOCART 2.0 score and the FAOS Sport/Rec subscale could be detected, indicating less impairments in sports activities in patients with a better incorporated osteochondral autograft.

Donor site morbidity has been reported to be the most common complication after AOT, occurring in 2–14% of patients [5, 10, 24, 25]. Interestingly, none of the patients in this study complained of pain or discomfort at the donor site at final follow-up. This unexpected finding may be attributed to the long follow-up period in this study, as previous research has shown that donor-site morbidity decreases with longer follow-up [8, 24].

The major strength of this study is the long follow-up period of 19.1 years on average. However, such a long follow-up period is accompanied by limitations such as a higher drop-out rate. Almost 63% of patients could not be followed up as they have changed residence or contact details. Further, no digital records are available from the inclusion period (i.e., 1997–2003). Therefore, patients could only be identified from the data in a handwritten logbook of the surgical procedures performed (i.e., date of surgery, type of surgery, name, date of birth, sex, and surgically treated side). Based on the available demographic data, no difference was observed between included patients and patients lost to follow-up. Given the limited sample size, it was not possible to perform adequately powered subgroup analyses. Future prospective studies should include control groups to identify the most appropriate treatment depending on the cartilage defect and thus facilitate an individualized management in the treatment of OLT.

## Conclusions

Autologous osteochondral transplantation is a safe and viable technique to treat OLT, achieving favorable clinical and functional outcomes without any donor-site morbidity and a 20-year survival rate of almost 80%. High patient satisfaction combined with an acceptable rate of limitations in activities of daily living and athletic and working performance underscore the clinical value of AOT. Higher athletic activity can be expected if the replaced cartilage has a more native appearance on MR imaging.
